# Acute intermittent hypoxia drives hepatic *de novo* lipogenesis in humans and rodents

**DOI:** 10.1016/j.metop.2022.100177

**Published:** 2022-03-14

**Authors:** Jonathan M. Hazlehurst, Teegan Reina Lim, Catriona Charlton, Jack J. Miller, Laura L. Gathercole, Thomas Cornfield, Nikolaos Nikolaou, Shelley E. Harris, Ahmad Moolla, Nantia Othonos, Lisa C. Heather, Thomas Marjot, Damian J. Tyler, Carolyn Carr, Leanne Hodson, Jane McKeating, Jeremy W. Tomlinson

**Affiliations:** a Oxford Centre for Diabetes, Endocrinology and Metabolism, NIHR Oxford Biomedical Research Centre, University of Oxford, Churchill Hospital, Oxford, OX3 7LE, UK; b Institute of Metabolism and Systems Research, University of Birmingham, Edgbaston, Birmingham, B15 2TT, UK; c Centre for Endocrinology, Diabetes and Metabolism, Birmingham Health Partners, Birmingham, B15 2TT, UK; d Department of Diabetes and Endocrinology, University Hospitals Birmingham NHS Foundation Trust, Birmingham, UK; e Department of Gastro & Hepatology, Singapore General Hospital, Outram Road, 544894, Singapore; f Department of Physiology, Anatomy and Genetics, University of Oxford, Oxford, OX1 3PT, UK; g Oxford Centre for Clinical Magnetic Resonance Research, Division of Cardiovascular Medicine, University of Oxford, Oxford, OX1 3PT, UK; h Department of Physics, Clarendon Laboratory, Parks Road, OX1 3PUT, Oxford, UK; i Department of Biological and Medical Sciences, Oxford Brookes University, Oxford, OX3 0BP, UK; j Nuffield Department of Medicine, University of Oxford, Oxford, OX3 7FZ, UK

**Keywords:** NAFLD, Hypoxia, HIF, Lipid metabolism

## Abstract

**Background and aims:**

Non-alcoholic fatty liver disease (NAFLD) is the most common chronic liver condition. It is tightly associated with an adverse metabolic phenotype (including obesity and type 2 diabetes) as well as with obstructive sleep apnoea (OSA) of which intermittent hypoxia is a critical component. Hepatic *de novo lipogenesis* (DNL) is a significant contributor to hepatic lipid content and the pathogenesis of NAFLD and has been proposed as a key pathway to target in the development of pharmacotherapies to treat NAFLD. Our aim is to use experimental models to investigate the impact of hypoxia on hepatic lipid metabolism independent of obesity and metabolic disease.

**Methods:**

Human and rodent studies incorporating stable isotopes and hyperinsulinaemic euglycaemic clamp studies were performed to assess the regulation of DNL and broader metabolic phenotype by intermittent hypoxia. Cell-based studies, including pharmacological and genetic manipulation of hypoxia-inducible factors (HIF), were used to examine the underlying mechanisms.

**Results:**

Hepatic DNL increased in response to acute intermittent hypoxia in humans, without alteration in glucose production or disposal. These observations were endorsed in a prolonged model of intermittent hypoxia in rodents using stable isotopic assessment of lipid metabolism. Changes in DNL were paralleled by increases in hepatic gene expression of acetyl CoA carboxylase 1 and fatty acid synthase. In human hepatoma cell lines, hypoxia increased both DNL and fatty acid uptake through HIF-1α and -2α dependent mechanisms.

**Conclusions:**

These studies provide robust evidence linking intermittent hypoxia and the regulation of DNL in both acute and sustained *in vivo* models of intermittent hypoxia, providing an important mechanistic link between hypoxia and NAFLD.

## Highlights

• Acute intermittent hypoxia increases hepatic *de novo lipogenesis* in humans *in vivo.*
• Prolonged intermittent hypoxia in rodent models increases hepatic *de novo lipogenesis.*
• The effect of hypoxia on *de novo lipogenesis* in cell lines is reliant on hypoxia-inducible factors.

## 1 Introduction

Non-alcoholic fatty liver disease (NAFLD) is the commonest worldwide cause of liver disease and is associated with increased mortality and risk of hepatocellular carcinoma (HCC) [1]. Hepatic *de novo lipogenesis* (DNL) is the synthesis of new fatty acids from acetyl coenzyme A (acetyl-CoA). DNL is estimated to contribute as much as 25% of the total hepatic lipid content [2] and DNL is increased in patients with NAFLD [3,4]. DNL is implicated in animal models of HCC [[5], [6], [7]] and both acetyl-Co A carboxylase (ACC) and fatty acid synthase (FASN), key enzymes of DNL, are increased in human HCC [[8], [9], [10]].

Obstructive sleep apnoea (OSA) is an independent risk factor for NAFLD [11], though underlying mechanism(s) linking OSA and hepatic steatosis are not well described. OSA is associated with intermittent hypoxia which increases hepatic triacylglycerol (TAG) levels in mouse models [12,13]. Mammalian cells adapt to low oxygen through an orchestrated transcriptional response regulated by hypoxia-inducible factors (HIFs) [14,15]. HIFs are heterodimeric transcription factors comprising a HIFα subunit (HIF-1α or HIF-2α) and a HIF-1β subunit and are regulated by oxygen-dependent and independent stress signals. HIFs control a wide range of genes involved in many cellular processes including energy metabolism and inflammation [16,17]. Mounting evidence reveals a role for HIFs in a number of diseases including cancer and inflammatory conditions, where pharmacological approaches to modulate HIF activity offer promising therapeutic avenues [18,19]. Genetic manipulation of HIFs or Von Hippel-Lindau (VHL) in knockout animals has confirmed a role for the hypoxic regulation of hepatic lipid metabolism though the relative role of DNL or fatty acid oxidation is uncertain [[20], [21], [22]].

Given the potential role of DNL in NAFLD pathogenesis we investigated the effect of intermittent hypoxia in an acute model in healthy volunteers in a proof-of-concept study using stable isotopes to track the fates of lipid and glucose within the context of a hyperinsulinemic euglycemic clamp. We have extended our findings into a rodent model with prolonged exposure to intermittent hypoxia. Finally, we have used human hepatoma cell lines, to assess whether our findings were reliant on the HIF signalling system. Our aim is to use these experimental models to investigate the impact of hypoxia on hepatic lipid metabolism independent of obesity and metabolic disease.

## 2 Material and methods

### 2.1 Human acute intermittent hypoxia protocol

This study was approved by the East Midlands Leicester South Research Ethics Committee (15/EM/0308). Nine healthy fasted male volunteers underwent a hyperinsulinemic-euglycemic clamp with concomitant infusions/oral stable isotopes in air prior to a repeat assessment 1 week later in conditions of acute intermittent hypoxia (AIH). Participants drank deuterated water (100%) the night before the assessments (3 g/kg of total body water in two divided doses) and water enriched to 3% *ad libitum* across the clamp to assess hepatic DNL via measurement of the incorporation of the deuterium label into the plasma TAG and VLDL-TAG [23,24]. Baseline enrichment of deuterated water in plasma water was corrected for prior to each study day. Infusion of stable isotopes began at the onset of the clamp ([^2^H]-glucose to measure endogenous glucose production rate and glucose disposal; [U^13^C]-palmitate to measure rates of lipolysis and lipid oxidation by the incorporation of ^13^C into exhaled CO_2_). Blood and breath were sampled across the clamp with sampling prior to the insulin infusion termed the “fasting” period and sampling at the end of the insulin infusion termed the “hyperinsulinemic” period. Subcutaneous abdominal adipose tissue microdialysis was used to measure glycerol release as a measured of lipolysis. After the initial 4-h basal period (“fasting”) the insulin and dextrose solution infusions began. This is the hyperinsulinemic-euglycemic clamp and this period lasted 2 h. The hyperinsulinemic-euglycemic clamp allows both the quantification of insulin resistance/sensitivity as well as to study the effect of insulin on the other metabolic processes examined. Glucose production and disposal was calculated using the modified Steele equations [25] on the basis of the tracer/tracee ratio (321/319; [2H]-glucose labelled/unlabeled glucose) measured using GCMS. NEFA appearance and disposal was calculated via GCMS as per previous studies [26,27]. Where AUC is presented, it is calculated using the trapezoidal method for the period (15 min) at the end of the basal period and the hyperinsulinemic period.

IH was achieved by controlling the FiO_2_ of the inspired gas. Volunteers wore a standard non-rebreathe mask with a T junction inlet connected to medical air (BOC, UK) and 5% oxygen; balance nitrogen (BOC Special Gases, UK). Volunteers were intermittently exposed to the hypoxic gas mix to achieve 12 desaturations/hour to 85–91% for 6 h. Full recovery to normal oxygen saturations were achieved between desaturations by exposing the participants to medical air and subsequently room air prior to the next desaturation. Reoxygenation commenced having reached nadir saturations. The total period of desaturation and reoxygenation lasted <2 min per hypoxic exposure.

### 2.2 Chronic intermittent hypoxia in rodents

Male Wistar rats were exposed to chronic intermittent hypoxia (CIH) or air for 14 days. During weekends animals were housed in ambient air. 16 rats were used in total with (8 exposed to CIH; 8 to normal ambient air). Animals were housed in groups of 4 males, with two cages in the hypoxic chamber and two control cages pair-housed directly below it to control from any stress arising from gas changes and/or handling. Animals were purchased via BioMedical Services, University of Oxford and were approximately 250g on arrival. Animals were allowed to acclimatize in the research facility for 6 days (Department of Human Physiology, Anatomy and Genetics (DPAG), University of Oxford). After 6 days of acclimatization and before any exposure to CIH, all animals underwent fasting bloods under isoflurane anaesthesia from the saphenous vein. Designed to mirror the intermittent hypoxia which is a feature of obstructive sleep apnoea the CIH took the form of 12 exposures/hour to a hypoxic gas mix for 6 h per day during the animals’ sleep period. The hypoxic gas mix was achieved by housing the animals in modified individually ventilated cages (IVCs) with inlet gases of nitrogen and oxygen. 32 s of nitrogen (0.5 bar pressure; ¼” internal diameter tubing) was sufficient to reduce the FiO_2_ in the cages to 10% O_2_ before the FiO_2_ was restored to 21% (FiO_2_ of air) by 4 s of oxygen (0.5 bar pressure; ¼” internal diameter tubing). Gas handling was performed at constant pressure with double-manifold solenoidal valves (Bürkert, DE) controlled via MOSFET relays using a Raspberry Pi (RS Components, UK) running custom software on Linux in soft real-time. The concentration of O_2_, CO_2_, and N_2_ within the modified IVCs was monitored using Normocap 200 Oxy (Datex Ohmeda/GE Healthcare, UK) gas analysers, controlled via a serial link, and linked to an automated warning and support system. Accuracy in the quantification of gas handling was ensured by the daily calibration of the Normocap 200 Oxy machines with Quick Cal™ Calibration Gas (GE Healthcare, UK). All gases were supplied by BOC, UK. Gas mixing through the IVC was aided by a small axial fan (40 mm × 40 mm x 10 mm). CO_2_ was removed within the chamber using adsorption with CarboLime (Allied Health Products, UK). Animals were fed normal chow (Harlan laboratories; with an Atwater Fuel Energy of 3.0 kcal/g, comprising 66% calories from carbohydrate, 22% from protein and 12% from fat). The drinking water was enriched to 3% with deuterated water (CK Isotopes, UK) for DNL assessment. To ensure tolerability of the CIH exposures animals were exposed to 2 h on day 1 of CIH; 4 h on day 2; and then 6 h for the following 14 days. Animals were sacrificed after a minimum IH exposure on the day of sacrifice of 2 h and no more than the 6-h protocol that the licence allowed. Animals were sacrificed in the fasted state by removing the heart in anaesthetised animals. Animals were weighed daily across the study protocol and assessed for markers of distress. Livers were dissected and plasma was taken for subsequent analysis.

### 2.3 Assessment of DNL in rodent model

#### 2.3.1 Stable isotope measurements

Drinking water was enriched to 3% with deuterated water throughout the protocol (CK Isotopes, UK). Livers of the rats were excised on termination and weighed and further dissected. 1.5 ml of methanol was added to the liver tissue (10 mg) and this was then sonicated for 5 min 3 ml of chloroform was then added. The TAG fraction was then isolated using the Folch method.

100 μl of plasma was aliquoted from each rat. Samples were taken at the beginning and end of the protocol. 200 μl of 0.9% NaCl was added. 3 ml of 2:1 chloroform:methanol solution was added. Given the low plasma volumes the plasma samples were not further separated to lipid fractions instead the whole sample was methylated prior to drying down and dissolving in chloroform. Samples were then run on GCMS (Agilent 5973, Agilent, UK) to identify the amount of labelled isotope (deuterated water) relative to the unlabeled metabolite in palmitate. % DNL was calculated using an estimated enrichment of the plasma water of 3% in keeping with the enrichment of the drinking water. Total TAG was also quantified within the liver (Triglyceride Colorimetric Assay kit, Cayman, Ann Arbor, USA) Total RNA was extracted from liver tissue using Tri-Reagent (Sigma-Aldrich, Dorset, UK) and concentration determined spectrophotometrically at OD260 on a Nanodrop spectrophotometer (Thermo Scientific, Hemel Hempstead, UK). Reverse transcription was performed in a 20 μl volume; 1 μg of total RNA incubated with 1 x RT Buffer, 100 mM dNTP Mix, 10x RT Random Primers, 50 U/μl MultiScribe Reverse Transcriptase and 20U/μl RNase Inhibitor. The reaction was carried out at 25 °C for 10 min, 37 °C for 120 min and terminated by heating to 85 °C for 5 min.

#### 2.3.2 Gene expression analysis

All quantitative PCR experiments were conducted on an ABI 7900HT (PerkinElmer Applied Biosystems, Warrington, UK). Reactions were performed in a 6 μl volume of 2 x Kapa Probe Fast Mastermix (Kapa Biosystems, Amsterdam, Netherlands). TaqMan assays (FAM labelled) and all reagents were supplied by Applied Biosystems (Applied Biosystems, Foster City, US). The reaction conditions were: 95 °C for 3 min, 40 cycles of 95 °C for 3 s and 60 °C for 20 s. The Ct of each sample was calculated using the following equation (where E is reaction efficiency determined from a standard curve): ΔCt = E^[min Ct-sample Ct]^ using the 1/40 dilution from a standard curve generated from a pool of all cDNAs as the calibrator. Relative expression ratio was calculated using the equation: ratio = ΔCt_[target]/_ ΔCt_[ref]_ and expression normalised to β-actin. Statistical analysis was performed on mean relative expression ratio values (Ratio = ΔCt[target]/ΔCt).

### 2.4 Human hepatoma cell culture models

Huh-7 (American Type Culture Collection, VA, USA) and HepG2 (Charles Rice, The Rockefeller University, New York, NY) hepatoma cells were maintained in Dulbecco's modified Eagle's medium (DMEM) (Gibco, USA), supplemented with 10% foetal bovine serum, 1% l-Glutamine, 1% non-essential amino acids and 50 units/ml penicillin/streptomycin (Gibco) in a humidified atmosphere at 37 °C, in 20% oxygen and 5% carbon dioxide. When the cells were 70–80% confluent, they were incubated under different oxygen tensions (1%, 3% or 21%) and 5% carbon dioxide with the balance of the atmosphere nitrogen) or treated with drugs for a further 24 h before RNA, protein and lipid extraction for the quantification of DNL and fatty acid uptake.

Drugs used in tissue culture models included NSC 134754 a HIF-pathway inhibitor (gift from Margaret Ashcroft, University College London, working concentration 0.02 μM) and FG4592 a PHD inhibitor (Cayman Chemicals, UK, working concentration 10 μM).

Full-length human HIF-1α and HIF-2α expression constructs (pCMVβ-HA-HIFα) were provided by Dr. Daniel Tennant (University of Birmingham, UK).

Cells were transfected with 8 μg of pHIF-1α or pHIF-2α per well using FuGENE™ 2000.24 h after transfection, cells were trypsinised and reseeded onto smaller wells for lipogenesis and FFA uptake as well as for protein/RNA analysis.

Cell lysates were prepared for western blotting. Briefly, 20 μg of protein lysates were loaded onto 8% sodium dodecyl sulphate-polyacrylamide gels (SDS-PAGE) and gels run at 200 V for 30 min. Proteins were transferred to polyvinylidene membranes (Millipore, USA) using a Mini *Trans*-Blot Electrophoresis Transfer System (Bio-Rad). Polyvinulidene membranes were cut to appropriate sizes to match the diameter of the gel and pre-treated with methanol for 2 min, rinsed with H_2_O and incubated in transfer buffer (25 mM Trizma Base, 0.2 M Glycine, 200 ml methanol and 10% SDS) at room temperature for 5 min. Gels were equilibrated in transfer buffer to prevent shrinking and transfer was carried out at 350 mA for 90 min at room temperature.

Following transfer to block non-specific antibody binding, membranes were incubated in antibody buffer (10 mM Trizma base, 0.1 M Sodium Chloride, 10% v/v Tween-20 and 5% Marvel dry milk) for 45 min at room temperature. The antibody blocking buffer was removed and the membranes were incubated in primary antibodies diluted with antibody buffer overnight with gentle agitation on a tube roller (Barloworld Scientific, UK) at 4 °C. Membranes were then washed 4 times for 5 min each (10 mM Trizma base, 0.1 M Sodium Chloride and 10% v/v Tween; pH 7.5). Incubation with HRP-conjugated secondary antibodies was carried out for 1.5 h at 4 °C followed by washing. Chemiluminescent detection of HRP-conjugated antibodies was achieved with an ECL Western Blotting Detection System (Amersham, UK). Membranes were incubated in ECL detection reagent for 1 min, wrapped in plastic and exposed to CL-Xposure X-Ray Films (Thermo Scientific) or using the PXi machine for 5–30 min.

### 2.5 Measures of lipid metabolism in cell culture

DNL was measured by the amount of uptake of 1-[^14^C]-acetate into the lipid component of cells, as we have described previously [28]. The ^14^C radioactivity retained in the cellular lipid was expressed as disintegrations per minute (dpm)/per well.

FFA-uptake was assessed using labelled palmitic acid. Cultured hepatoma cells were treated in 24-well plates and incubated with 500 μl of serum free media containing 0.1 mmol/L palmitate (9,10-[^3^H] palmitic acid (5uCi/ml) (GE Healthcare, Bucks, UK) with cold palmitate to a final concentration of 10 μM palmitate, 2% BSA and treatments for 24 h. After incubation, cells were washed with cold PBS three times before 25 μl of 1% Triton was added. Scraped cells were transferred to scintillation vials with scintillation cocktail to measure NEFA uptake. This assay measures the intracellular (cytosolic) accumulation of 9,10-[^3^H]-labelled palmitate tracer. After incubation intracellular lipids are extracted and the retained ^3^H radioactivity is measured by scintillation counting.

Rate of β-oxidation was measured by the conversion of 9,10-[^3^H]-palmitate (PerkinElmer) to ^3^H_2_O, using a modification of our previously published method [28]. After incubation, the 250 μl of media was retained and precipitated twice with equal volumes of 10% trichloroacetic acid to remove excess labelled palmitate. The supernatants (0.5 ml) were extracted by addition of methanol:chloroform (2:1) and 1 ml of 2 mol/L KCl:HCl, followed by centrifugation at 3000*g* for 5 min. Aqueous phase (0.5 ml) was added to scintillation cocktail (PerkinElmer, Bucks, UK), Aqueous samples were counted using a Wallac 1414 Liquid Scintillation Counter (PerkinElmer, Bucks, UK) to measure the rate of β-oxidation.

Quantitative PCR (qPCR) in the cell-based models was carried out using Applied Biosystems reagents and expression assays (Qiagen). qPCRs for genes of interest and for housekeeping gene GAPDH were carried out in singleplex (i.e. reactions carried out in separate wells). For the gene of interest in a single reaction (100 wells) the following components were added: 750 μl of 2X PCR Mix, 25 μl GAPDH, 30 μl RT-Tag enzyme mix and 400 μl nuclease free water. 2 μl of RNA sample was added into each well. Samples were run using 7500 real-time PCR system (Applied Biosystems, Warrington, UK). Data were expressed as ct values (ct = cycle number at which logarithmic PCR plots cross a calculated threshold line) and used to determine Δct values [Δct = (ct of the target gene) – (ct of the housekeeping gene)], lower Δct values reflecting higher mRNA expression. Fold changes were calculated using transformation [fold increase = 2-difference in ΔCT].

### 2.6 Statistical analysis

Data is presented throughout as mean ± standard error with Student's t tests used for between group comparison. Individual data points are included within each figure to ensure transparency. Where AUC is presented, it is calculated using the trapezoidal method.

## 3 Results

### 3.1 Acute intermittent hypoxia increases DNL in healthy human volunteers

**Fig. 1:**
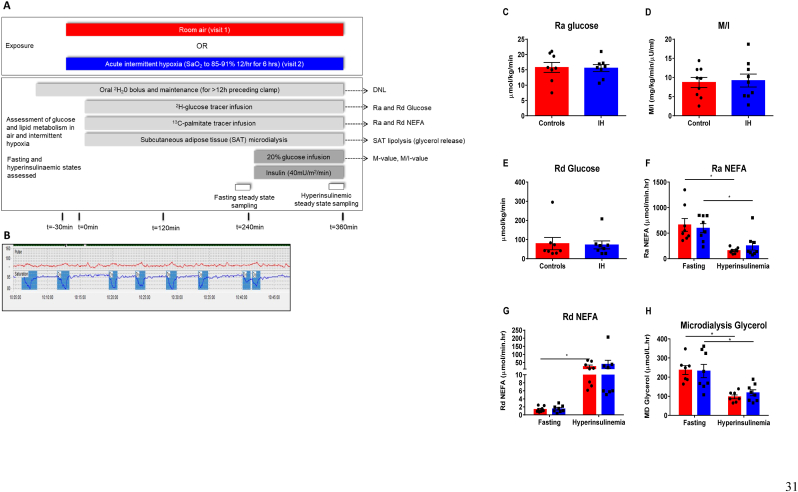
The assessment of the effect of intermittent hypoxia in the fasting state and during a hyperinsulinemia euglycemic clamp on insulin sensitivity, glucose metabolism (C–E) and free fatty acid metabolism (F–H) in healthy male volunteers. A: The experimental protocol comparing the effect of acute intermittent hypoxia (blue bars) and room air (red bars) (DNL = *de novo* lipogenesis; SAT = subcutaneous adipose tissue). B: A representative trace of continuous SaO_2_ and heart rate monitoring showing the effect of the intermittent hypoxia protocol to achieve desaturations (using ApneaLink^TM^ plus). C–F: The assessment of the effect of intermittent hypoxia (blue bars) vs control (air) (red bars) on indices of glucose and lipid metabolism measured using a combination of stable isotopes and subcutaneous abdominal adipose microdialysis. C: Glucose production was quantified using the appearance of isotopically labelled glucose ([^2^H]-glucose) measured in the fasting steady state. D: The rate of glucose infusion required to achieve euglycemia corrected for plasma insulin (M/I) measured in the hyperinsulinemia steady state. E: Glucose disposal was quantified isotopically in the hyperinsulinemia state. F: NEFA appearance. G: NEFA disposal. NEFA metabolism (F & G) was quantified by measuring the appearance and disposal of isotopically labelled palmitate ([U^13^C]-palmitate) within the NEFA total palmitate isolated from the plasma fraction measured using gas chromatography mass spectrometry. H: The rate of glycerol appearance within the subcutaneous abdominal adipose tissue interstitial fluid assed by adipose tissue microdialysis in the fasting and hyperinsulinemia steady state. Data are presented as mean ± standard error, *p < 0.05 (Student's *t*-test). (For interpretation of the references to colour in this figure legend, the reader is referred to the Web version of this article.)

We established an experimental medicine, study in healthy male volunteers (n = 9) which was approved by East Midlands Leicester South Research Ethics Committee (15/EM/0308). Volunteers underwent a hyperinsulinemic-euglycaemic clamp under both normoxia and AIH (12 desaturations/hour to a pulse oximetry saturation (SaO_2_) of 85–91%). The total exposure to AIH was 6 h; sampling was undertaken in both the fasted state (after 4 h of the exposure) and after 2 h of a hyperinsulinemic-euglycemic clamp (after 6 h of exposure) [Fig. 1A]. Stable isotope tracers (^2^H_2_O, ^2^H-glucose, and ^13^C-palmitate) allowed the measurement of both glucose and FFA flux, as well as DNL. The AIH protocol was well tolerated by participants and resulted in a rapid reduction and resolution in the oxygen saturations [Fig. 1B] with a mean of 11.3 ± 0.2 desaturations/h to a nadir pulse oximetry O_2_ saturation of 86.7 ± 0.7%.

Glucose production rate (Ra Glucose) was not altered by exposure to AIH (15.8 ± 4.6 *vs*. 15.5 ± 3.2 μmol/kg/min, air *vs.* AIH, p = 0.91) [Fig. 1C]. The rate of glucose infusion required to maintain euglycemia during hyperinsulinemia (M-value) was also unchanged (7.7 ± 3.8 *vs.* 8.5 ± 4.7 mg/kg/min, air *vs.* AIH, p = 0.10) after correcting for plasma insulin levels (M/I-value) (8.7 ± 3.9 *vs*. 9.2 ± 5.0 mg/kg/min/μU/ml, air *vs*. AIH, p = 0.54) [Fig. 1D]. Stable isotope measurements of glucose disposal (Gd) were also unaffected (79.0 ± 89.6 *vs.* 72.0 ± 58.2 μmol/kg/min, air *vs.* AIH, p = 0.639) [Fig. 1E].

Whole body fatty acid turnover was assessed using a^13^C-palmitate infusion. Hyperinsulinemia suppressed the rate of non-esterified fatty acid (NEFA) appearance (Ra NEFA) and increased NEFA disposal (Rd NEFA) consistent with the suppression of adipose tissue lipolysis. AIH had no effect on NEFA turnover (Ra NEFA: (fasting) 661 ± 353 *vs.* 598 ± 245; (hyperinsulinemia) 158 ± 54 *vs.* 254 ± 241; Rd NEFA: (fasting) 1.4 ± 0.67 *vs.* 1.5 ± 0.84; (hyperinsulinemia) 25 ± 24 *vs.* 39 ± 69 μmol/min.hr (air *vs.* AIH) [Fig. 1F and G]. Adipose tissue microdialysis was used to quantify lipolysis (measured by glycerol release), within the abdominal subcutaneous adipose tissue (SAT) depot. As with the whole-body measurement of lipolysis, insulin suppressed SAT glycerol release, but AIH had no effect [Fig. 1H].

**Fig. 2:**
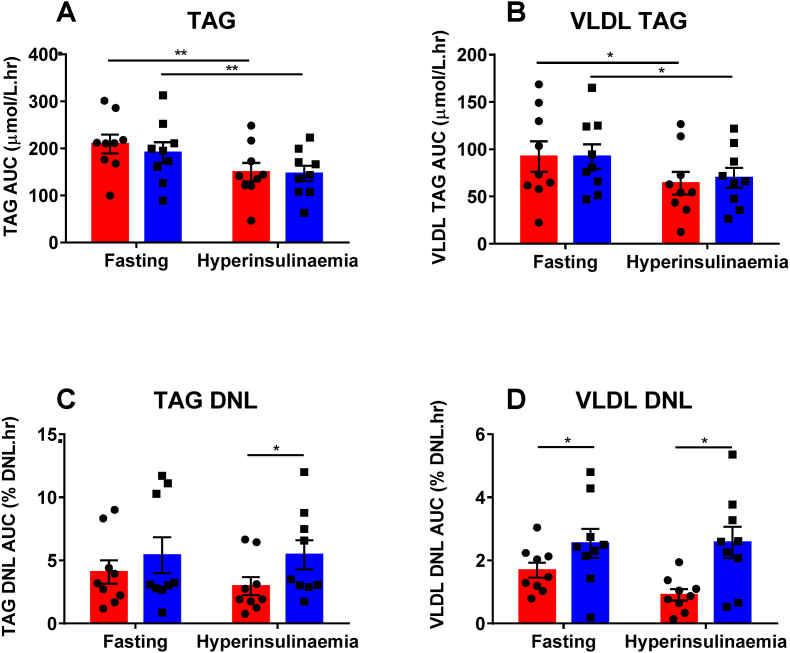
**The effect of acute intermittent hypoxia on plasma TAG, VLDL TAG and DNL measured in the plasma TAG and VLDL TAG fractions in healthy male volunteers**. Room air (red bars), intermittent hypoxia (blue bars). A and B: TAG was measured within the plasma (A) and isolated VLDL fractions (B). C and D: DNL measured in the plasma TAG (C) and VLDL TAG fractions (D). DNL was measured via quantification of deuterated water into the isolated palmitate TAG fraction either from whole plasma (C) or the liver specific VLDL fraction (D). (VLDL = very low-density lipoprotein; TAG = triacylglycerol; DNL = *de novo* lipogenesis); Data is presented as mean ± standard error, *p < 0.05 (Student's *t*-test). (For interpretation of the references to colour in this figure legend, the reader is referred to the Web version of this article.)

Total plasma TAG and VLDL-TAG decreased during hyperinsulinemia consistent with insulin-mediated suppression of VLDL export from the liver [29]. This was unchanged by AIH [Fig. 2A and B]. However, there was a significant increase in DNL in both the fasting state (VLDL-TAG incorporation only) and during hyperinsulinemia (both total plasma TAG and VLDL-TAG) following AIH [Fig. 2C and D].

### 3.2 Rodents exposed to intermittent hypoxia have increased hepatic DNL

**Fig. 3:**
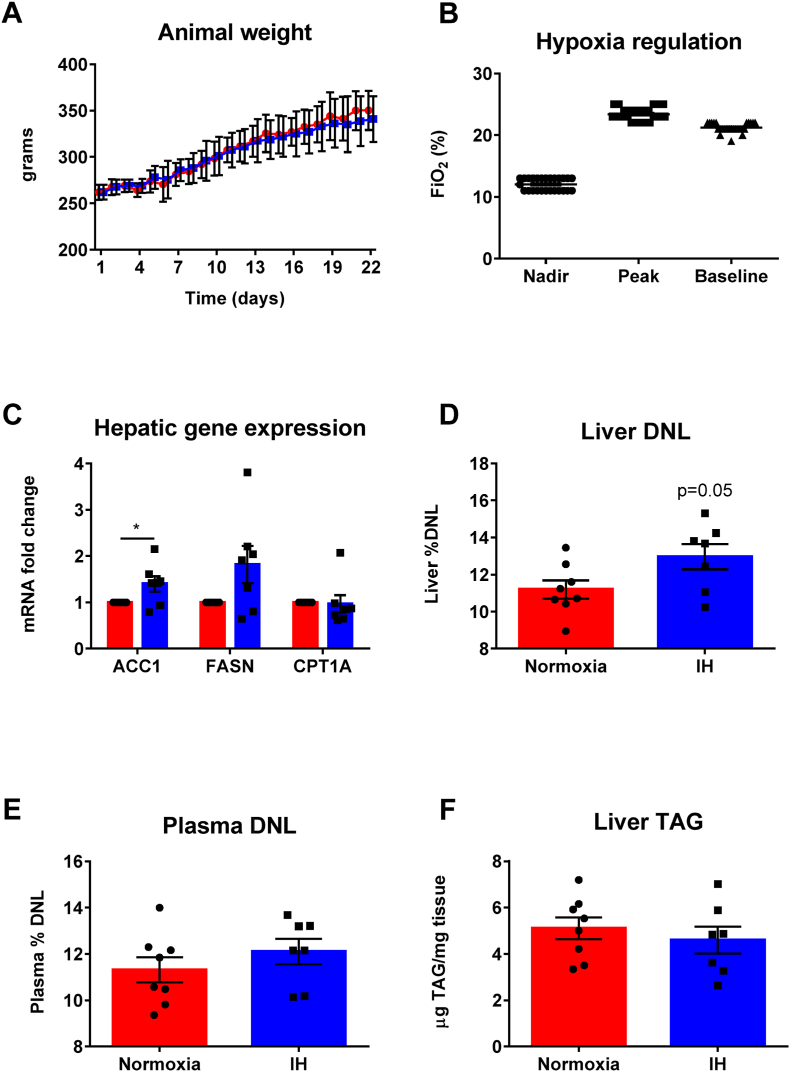
**The effect of intermittent hypoxia on DNL in rodents**. Adult male Wistar rats were exposed to either normoxia (ambient air) (n = 8) (red bars) or intermittent hypoxia (IH) (blue bars) (n = 7) (FiO_2_ to 10 ± 1% 12 times/hr for 6 h/day during sleep for 14 days). A: Animal weights across the study protocol (circles (red) = controls; squares (blue) = intermittent hypoxia). B: Nadir, peak and baseline oxygenation of the cages (representative data from 1 h in 2 cages). C: Hepatic gene expression (*ACC1*: acetyl-coA carboxylase; *FASN*: fatty acid synthase; *CTP1A*: carnitine palmitoyltransferase 1A). D: Liver DNL measured as enrichment of the liver lipid palmitate fraction with deuterated water (271/270 = tracer:tracee ratio of enriched to non-enriched palmitate). E: Plasma DNL measured as enrichment of the plasma lipid palmitate fraction with deuterated water. F: Liver triaclygylercol (TAG). Control animals in air (red bars), intermittent hypoxia (IH) (blue bars). Data is presented as mean ± standard error, *p < 0.05 (Student's *t*-test). (For interpretation of the references to colour in this figure legend, the reader is referred to the Web version of this article.)

Having demonstrated the effect of acute intermittent hypoxia on DNL in our human study we sought to examine the effect of more sustained intermittent hypoxia exposure in rats by exposing adult male Wistar rats, fed a standard chow diet, to prolonged intermittent hypoxia (12 exposures/hour to FiO_2_ 10% for 6 h/day during the animals’ sleep period) (n = 7) or normal room air (n = 8). All animal procedures were performed in accordance with relevant UK legislation (ASPA 1986) following an explicit ethical review process and undertaken within institutional and national guidance. The protocol was well tolerated with no signs of animal distress or change in body weight when compared to normal room air [Fig. 3A]. The gas-handling system was able to reliably and rapidly reduce the FiO_2_ to 10 ± 1% and to subsequently restore FiO_2_ to 21% to achieve 12 exposures/hour without deviation in the CO_2_ or N_2_, in accordance with the protocol and animal licence [Fig. 3B]. The gas system and animal behavior were continuously monitored by an investigator when in conditions of intermittent hypoxia.

Hepatic mRNA expression of the rate liming enzymes for DNL, *FASN* and *ACC*, increased following prolonged intermittent hypoxia. Transcript levels of carnitine palmitoyltransferase 1A (*CPT1A*), a regulator of FFA oxidation were unchanged [Fig. 3C]. Liver % DNL (measured within liver tissue) but not plasma % DNL increased in intermittent hypoxia (p = 0.05) [Fig 3D; Fig 3E]. Liver weight, histological assessment for steatosis (data not shown) and hepatic TAG were comparable between conditions of intermittent hypoxia and normoxia, potentially reflecting the relatively short duration of intervention [Fig. 3F].

### 3.3 Hypoxia regulates *de novo* lipogenesis in human hepatoma cell models

Having demonstrated the impact of hypoxia on DNL in both human and rodent models, we next explored the cellular mechanisms underpinning our observations in human hepatoma models (Huh-7 and HepG2).

**Fig. 4:**
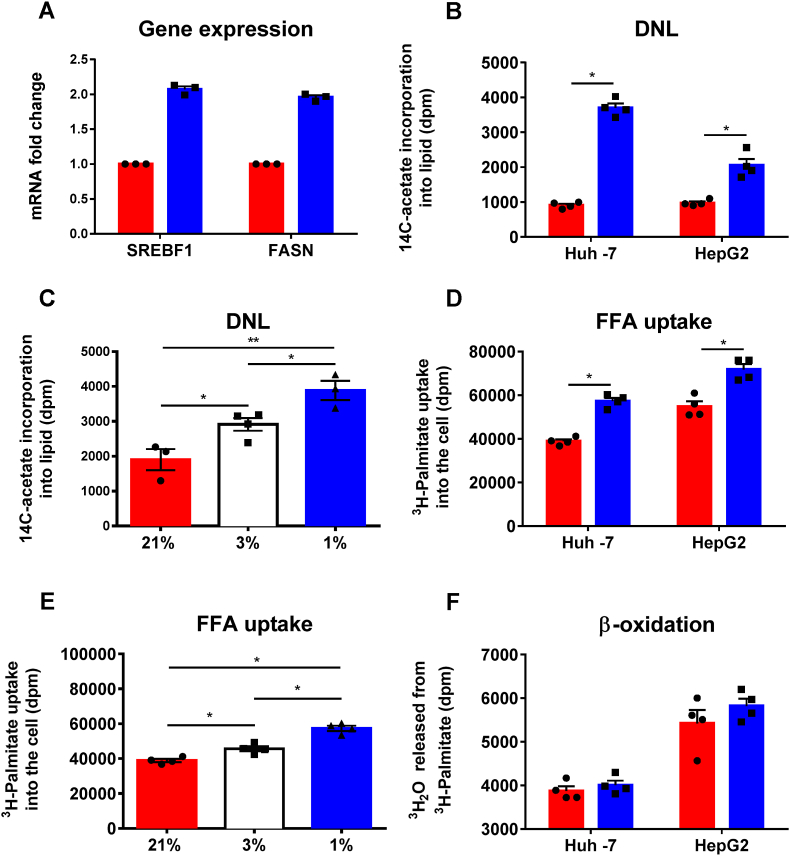
**The effect of hypoxia on *in* vitro hepatocyte lipid metabolism.** A: mRNA levels measured by qRT-PCR in Huh-7 hepatoma cells incubated under 1% (blue bars) or 21% (red bars) oxygen for 24 h. Gene expression of (sterol regulatory element-binding protein 1 (*SREBF1*) and fatty acid synthase (*FASN*), relative to *GAPDH*. B: The effect of hypoxia (1% (blue bars) vs 21% (red bars)) for 24 h on *de novo lipogenesis* in Huh-7 and HepG2 cells (DNL). C: The effect of hypoxia ((1% (blue bars), 3% (white bars) and 21% (red bars)) on DNL (Huh7 cells). DNL was determined by measuring 1-[^14^C]-acetate incorporation into the lipid fraction of cells incubated with ^14^C-acetate cultured under stated oxygen tensions. D: The effect of hypoxia (1% (blue bars) vs 21% (red bars)) for 24 h on free fatty acid (FFA) uptake in Huh7 and HepG2 cells. E: The effect of hypoxia ((1% (blue bars), 3% (white bars) and 21% (red bars)) on FFA uptake (Huh7 cells). FFA uptake was defined by the amount of ^3^H palmitate taken up by cells after 12 h incubation with ^3^H-palmitate in serum-free media F: The effect of hypoxia on β-oxidation (1% (blue bars) vs 21% (red bars)) for 24 h in Huh-7 and HepG2 cells. β-oxidation was measured by the amount of ^3^H-water released by cells into the culture media. Data represents 3 independent experiments in quadruplicates. Error bars indicate mean ± standard error (n = 3) *p < 0.05; **p < 0.001 (Student's t-test). (For interpretation of the references to colour in this figure legend, the reader is referred to the Web version of this article.)

Hypoxia (1% O_2_, 24 h) exposure increased mRNA levels of sterol regulatory element-binding protein 1 (*SREBF1*), fatty acid synthase (*FASN*) in Huh-7 cells [Fig. 4A]. To assess the functional impact on DNL, we measured 1-[^14^C]-acetate incorporation into the cellular lipid fraction in Huh-7 and HepG2 cells. Hypoxia caused a 4-fold and 2-fold increase in DNL in Huh-7 and HepG2 cells respectively [Fig. 4B]. There was a close association between DNL and oxygen tension (21% oxygen: 1901 ± 526; 3% oxygen 2916 ± 367, *p* = 0.029; 1% oxygen 3888 ± 482 dpm, *p* = 0.009, *p-*values compared to 21% oxygen) [Fig. 4C]. ^3^H-Palmitate was used to assess FFA uptake [30]. Hypoxia increased FFA uptake in both Huh-7 and HepG2 cells [Fig. 4D]. Hypoxia increased FFA uptake in Huh-7 cells (21% oxygen: 38875 ± 1821; 3% oxygen: 45645 ± 2810, *p* = 0.0068; 1% oxygen: 57380 ± 2935 dpm, *p* < 0.0001) [Fig. 4E]. The generation of ^3^H_2_O from ^3^H-palmitate provides a marker of cellular lipid oxidation, however there was no effect of hypoxia on β-oxidation [Fig. 4F].

### 3.4 A role for HIFs to regulate *de novo* lipogenesis

**Fig. 5:**
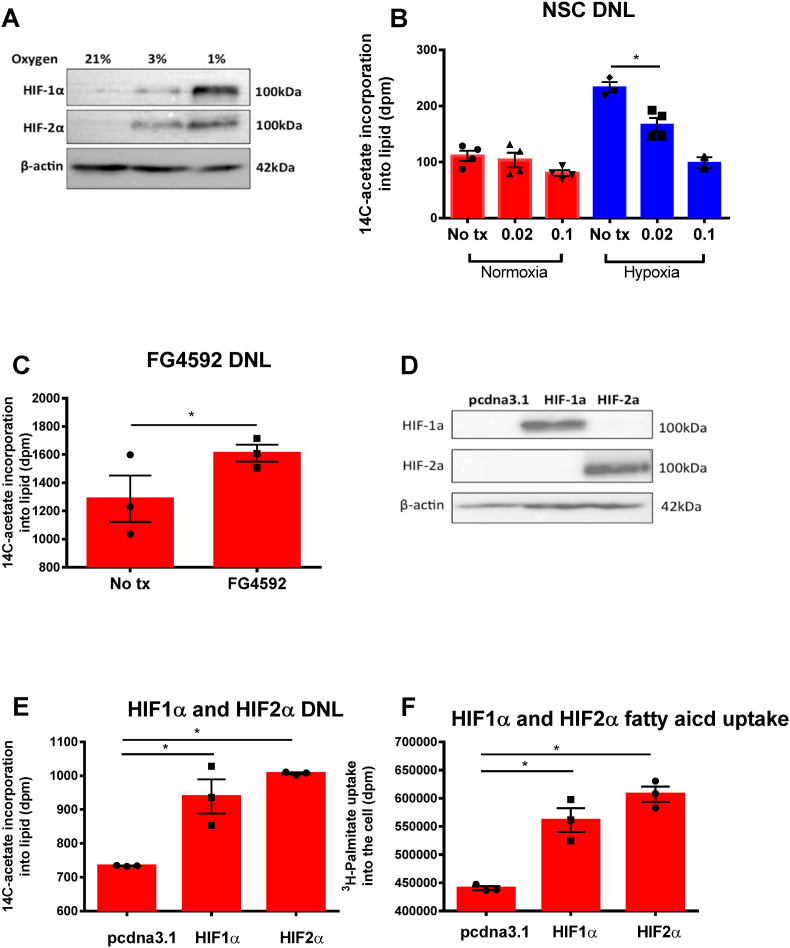
**The hypoxia induced effect on *de novo lipogenesis* is HIF dependent.** A: HIF1-α and HIF2-α Western blot in response to different oxygen tensions. B: Inhibiting HIF signaling with NSC 134754 (NSC) abrogates the hypoxia (1% (blue bars), 21% (red bars)) induced increase in DNL in a dose-dependent manner (NSC doses are 0.02–0.1 μM). C: HIF stabilization with 10 μM of FG4592 results in increased DNL in 21% oxygen (red bars). D: HIF1-α and HIF2-α can be stably overexpressed in normoxia. E: Overexpression of HIF1-α and HIF2-α results in increased DNL and fatty acid uptake (F). Huh-7 cells were transfected with siRNA-mediated scrambled RNA (scRNA), HIF1 or HIF-2α for 24 h. Cells were incubated at 21% (red bars) or 1% (blue bars) oxygen for a further 24 h. Following this, HIF protein expression was determined by Western blotting from lysate. Following transfection, 1-[^14^C]-acetate incorporation into lipid and ^3^H-palmitate uptake was measured in these cells. Experiments were performed three times in quadruplicates. Data are presented as mean ± standard error, *p < 0.05; **p < 0.001 (Student's *t*-test). (For interpretation of the references to colour in this figure legend, the reader is referred to the Web version of this article.)

Culturing Huh-7 cells under hypoxic conditions stabilised HIF-1α and HIF-2α isoforms [Fig. 5A]. The emetine analogue, NSC 134754 (NSC) is known to inhibit HIF-signaling and limits HIF expression under hypoxic conditions [31]. Here, the hypoxia-dependent (1%, 24 h) increase in DNL was reversed by treating Huh-7 cells with NSC (0.1 μM, 24 h) [Fig. 5B]. Under hypoxia, there was a dose-dependency of NSC to limit hypoxia-induced DNL, additionally NSC had no effect on DNL in normoxia consistent with limited off target effects [Fig. 5B]. NSC had no effect on cell viability at concentrations up to, and including 0.1 μM for 24 h (data not shown). To further validate a role for HIFs in regulating DNL, we treated cells with a HIF prolyl hydroxylase inhibitor, FG4592, that stabilises HIF expression under normoxic conditions [32]. FG4592 increased DNL [Fig. 5C]. Further experiments to over-express HIF-1α and HIF-2α in Huh-7 cells [Fig. 5D] resulted in an increase in DNL compared to cells transfected with vector alone (pcdna3.1: 734 ± 1.5; HIF-1α 939 ± 88, *p* = 0.015; HIF-2α 1007 ± 4.5 dpm, *p* = 0.0001) [Fig. 5E]. Furthermore, FFA uptake was increased in both HIF-1α and HIF-2α overexpressing models (pcdna3.1: 440564 ± 6000; HIF-1α 561311 ± 36826, *p* = 0.005; HIF-2α 607512 ± 23734 dpm, *p* = 0.0003) [Fig. 5F]. Taken together, these data from the pharmacological and genetic manipulation of HIFs demonstrate a role for HIFs in regulating DNL and fatty acid uptake.

## 4 Discussion

We have demonstrated that exposure to intermittent hypoxia is associated with an increase in hepatic DNL in humans and rodent models. These findings are endosed in human hpetoma cell lines where teh impact of hypoxia is critically dependent upon HIF stabilization.

Using a combination of cellular, rodent and experimental medicine studies, we have shown a functional increase in DNL in response hypoxia that within our cellular models was HIF-dependent. Previous *in vitro* studies have examined the effect of sustained hypoxia on hepatocellular lipid metabolism by studying changes in gene expression [33,34] or measuring intracellular TAG [34,35]. It has been suggested that intracellular lipid accumulation may arise as a result of reduced β-oxidation as well as increased FFA uptake rather than increased DNL [[33], [34], [35]]. Our data show a role for hypoxia to regulate FFA uptake in different hepatocyte models and is dependent on oxygen tension as previously reported [33]. Although previous studies suggested a decrease in β-oxidation [34,35] our functional assessment of β-oxidation was not altered under *in vitro* hypoxic conditions. Our *in vitro* studies provide robust evidence for HIFs to regulate DNL and are in agreement with published gene expression data [36].

DNL can be assessed using stable isotopes or by measuring surrogate ratios of FFA in the TAG or VLDL-TAG lipid fraction. Although DNL contributes only ≈25% of the hepatic lipid content measured in patients with NAFLD [2], the finding of increased rates of DNL in patients with elevated liver fat [3,4] are suggestive of a pathogenic role. Regions of steatohepatitis and cirrhosis in liver biopsies are enriched with saturated TAG (typically a product of DNL) suggesting an important role for DNL in NAFLD severity [37]. Given that steatosis increases with decreasing oxygen saturation across the hepatic porto-central oxygen [38] there is further plausibility for a role of hypoxic regulation of DNL in NAFLD pathogenesis. In our experimental models (cellular and *in vivo*) we did not see changes in β-oxidation of NEFA, yet given the importance of hypoxic regulation of β-oxidation within liver zonation [39] both processes may occur in parallel.

Animal data has shown an increase in lipogenic hepatic gene expression (*SREBF1* and *ACC1*) as well as hepatic TAG [12] following acute exposures in lean animals [13]. Intermittent hypoxia also results in hepatic inflammation [40] which is likely to promote the progression of hepatic steatosis to steatohepatitis. A limitation of many published studies is that variable oxygen availability has been studied alongside an obesogenic diet and dissecting the specific contribution of intermittent hypoxia to DNL and hepatic lipid accumulation has not been possible. Furthermore, the models employed by most studies include longer periods of hypoxia (often more than 30 desaturations/hour) covering a more substantial portion of a 24-h period than typical sleep duration in a patient with OSA (rodent studies typically involve 8–10 h of intermittent hypoxia/day). Finally, the use of stable isotope methodologies within our studies affords a more dynamic assessment of metabolic pathways and lipid flux.

OSA is associated with morbidity [41] and mortality [42]. Dissecting the components that drive this adverse outcome is confounded by co-existing co-morbidities e.g. obesity, type 2 diabetes and cardiovascular disease. However, even in people without obesity the nadir peripheral oxygen saturations of patients with OSA positively associates with the presence and severity of NAFLD [43]. Consistent with this, a recent meta-analysis confirmed the association between OSA and NAFLD [11]. In a study of patients undergoing bariatric surgery, oxygen desaturation associated with indices of severe NAFLD on biopsy but not with subcutaneous or visceral adipose morphology [44]. This mirrors our *in vivo* findings in which AIH had an effect on hepatic DNL but no effect on adipose tissue lipolysis (globally or specifically within the abdominal subcutaneous depot). We have demonstrated that intermittent hypoxia can drive DNL *in vitro* and *in vivo,* providing a potential mechanism linking hypoxia and NAFLD.

Our approach does have some specific limitations. We have used male rodents and adult men in our experimental studies and though fasting DNL appears comparable between men and women there may be differences in some experimental conditions [45] and we cannot simply assume that observations in men translate directly to women. Within our clinical study, the lack of impact on glucose handling might have been a reflection of the relatively short duration of AIH, however within the ethical constraints of a healthy volunteer, proof-of-concept study, we were unable to justify more prolonged or repeated exposure. Within our rodent and *in vitro* studies with hindsight we may have examined a more complete panel of genes regulating DNL as well as protein expression. Additionally future work may examine in greater detail potential changes to fatty acid transport. In addition, within our human studies the participants lacked many of the comorbidities associated with NAFLD and OSA, however, despite this were able to show and independent effect of hypoxia on DNL *in vivo*.

## 5 Conclusions

We have demonstrated that AIH increases hepatic *de novo lipogenesis* (DNL) in human healthy volunteers. Rodent and cellular studies have both endorsed our findings and shown clear HIF dependance. Our studies not only highlight the importance of DNL, but also suggest that we need to carefully delineate the precise mechanisms contributing to the development of NAFLD (which will be different in individual patients), such that we can be in a position to tailor future therapies in a precise and personalized manner.

## Author contributions

JMH, JWT, LH, TRL and JM conceived the studies. JMH performed the rodent and human experiments. TRL performed the cell-based studies with technical assistance of LLG and under the supervision of JAM. C Charlton and TC undertook the mass spectrometry analysis of glucose turnover and provided significant mass spectrometry support for the other aspects of the *in vivo* work to JMH with additional support and guidance of LH. JJM designed and constructed the intermittent hypoxia automation for the rodent study and provided technical expertise and assistance throughout the rodent study. LLG, NN, SEH, AM, TM, C Charlton and TC supported JMH in analyzing samples from the rodent and human studies. C Carr, DJT, JJM, and LCH provided oversight and support of the rodent work. JMH, JWT, TRL, and JAM took the leading role in writing the manuscript and designing the figures. All authors contributed to the editing and proofreading of the final draft.

## Financial support

The work was supported as follows: 10.13039/501100000265Medical Research Council (program grant to JWT ref. MR/P011462/1; Wellcome Trust IA 200838/Z/16/Z to JAM; Wellcome Trust CRTF to JH ref. 104458/Z/14/Z; CRTF to RL ref. MR/J010561/1); 10.13039/501100013373NIHR Oxford Biomedical Research Centre (principal investigator award to JWT); and 10.13039/501100000274British Heart Foundation (senior fellowship to LH ref. FS/15/56/31645;; DJT FS/14/17/30634) and an EPSRC Doctoral Prize Fellowship (ref. EP/M508111/1) and Novo Nordisk Postdoctoral Fellowship awarded to JJM). The views expressed are those of the author(s) and not necessarily those of the NHS, the NIHR or the Department of Health.

## Declaration of competing interest

None of the authors have any conflicts of interest or any relevant financial disclosures.
